# Mechanistic contribution of CaV3.2 calcium channels to trigeminal neuralgia pathophysiology not clarified

**DOI:** 10.12688/f1000research.122997.1

**Published:** 2022-06-29

**Authors:** Wolfgang Liedtke

**Affiliations:** 1 Department of Molecular Pathobiology - Dental Pain Research, New York University College of Dentistry, New York, NY, 10016, USA; 2 Department of Neurology/ Headache-Pain, Duke University Medical Center, Durham, NC, 27710, USA

**Keywords:** trigeminal neuralgia - CaV3.2 ion channel - channelopathy - pain - CACNA1H gene - familial trigeminal neuralgia

## Abstract

Trigeminal neuralgia (TN) is a rare, yet debilitating trigeminal pain disorder, with jolts of supramaximal-debilitating pain in one or more of the three trigeminal branches. Familial TN is now recognized, with a recent report describing several human genetic polymorphisms. One affected gene is the voltage-gated calcium channel, CaV3.2 ( *CACNA1H*), with 19 polymorphisms first described. A recent study in PAIN by Gambeta-et-al (DOI:10.1097/j.pain.0000000000002651) is entitled " *CaV3.2 calcium channels contribute to trigeminal neuralgia* ". Here, I call into question their claim. My main arguments are 1)-3):  1) Gambeta-et-al studied 4/19 mutations reported in heterologous cellular expression, with two mutations showing gain-of-function of CaV3.2, two mutations not showing gain-of-function. Therefore the exemplary picks of familial TN-associated CaV3.2 mutations do not show a uniform change of channel function, such as gain-of-function.  2) In Gambeta-et-al, one gain-of-function mutation, CaV3.2(G563R) was directed to mouse trigeminal ganglion (TG) neurons, and their resulting hyperexcitability was demonstrated. A critical control of a non-gain-of-function channel was not included here, it was unclear whether neurons were separated by sex, and human sensory neurons were not used. Importantly, it is not clear that TG  neurons are the critical cellular site of CaV3.2 mutations. 3) Gambeta-et-al used CaV3.2-/- pan-null knockout mice. Human TN-associated CaV3.2 mutations were not over-expressed. They used a infraorbital nerve constriction injury and measured facial heat hyperalgesia.  CaV3.2-/- show a pain phenotype similar to control, yet are not affected by a CaV3-inhibitory compound, Z944. My argument is that when starting with TN-associated human mutations, use of a trigeminal neuropathic pain model is of limited value, and that human mutations have to be expressed against a mouse null background. Re thermal cue, Gambeta-et-al failed to study cold-evoked pain which is a TN clinical hallmark.

Thus, Gambeta-et-al's 2022 PAIN-paper offers little new mechanistic evidence why CaV3.2 polymorphisms are associated with trigeminal neuralgia.

In “ *CaV3.2 calcium channels contribute to trigeminal neuralgia*” the authors state “ *TN is a debilitating syndrome […] Understanding the pathophysiology of TN is needed to provide new targets for therapeutic intervention.*” ( Gambeta *et al*. 2022).—No disagreements here.

Whether their recent paper ( Gambeta *et al.* 2022) indeed makes constructive progress on this front as claimed should be open for discussion. In this *Correspondence* I am asking for readers’ consideration whether data as reported and authors’ discussion of these data can indeed support their claim. In view of Dong *et al*. (2020), where polymorphisms in the *CACNA1H* gene were reported by Dong *et al*. as associated with familial trigeminal neuralgia, the lead question of “And what is new?” has to be asked, with particular focus on how findings by Gambeta *et al*. provide new mechanistic understanding of this debilitating pain condition in affected families. In other words, if a *CACNA1H* polymorphism carrier patient with trigeminal neuralgia asks their doctor “So tell me, doctor, why do I—and my family members—have these terrible jolts of pain, how does it really work?”; can they get a more rationally-based answer as a result of Gambeta *et al*.’s study?

Presented data appear the result of dedicated physiologic, animal behavior, neurocellular, molecular biology and biochemical experimentation. However, results as presented simply to do not support the tendered claims and conclusions.

Gambeta *et al*. has three subsections, (i) directed expression of mutant CaV3.2 channels in heterologous cellular systems, (ii) directed expression of CaV3.2(G563R) in trigeminal ganglion sensory neurons, (iii) mouse studies using CaV3.2−/− pan-null knockout mice and a CaV3-selective chemical inhibitor, Z944. With regards to (i), there is non-congruence whether there is gain-of-function of the interrogated variants, or not. For (ii), the assumption that trigeminal ganglion sensory neurons are the critical cellular players in trigeminal neuralgia of CaV3.2 polymorphisms is unfounded. Only one variant is tested, not in human sensory neurons. With (iii), a model for trigeminal neuropathic pain after peripheral branch injury is used, and CaV3.2 mutations are not tested.

Thus, in terms of pathophysiology of trigeminal neuralgia we are left where we were before publication of Gambeta *et al*.’s recent paper in PAIN, perhaps scratching our heads more re the roles of CaV3.2 ion channels.

With regards to some critical detail:

The human genetic findings from Dong *et al.* (2020) are the starting point. As ion channels with some supporting evidence to their role in pathologic pain, CaV3.2 channels are legitimate targets for exploration of their mechanistic contribution to trigeminal neuralgia. How did the authors arrive at the four variants selected, out of a choice of 19? Ideally all variants should be interrogated by a validated first-pass screen, which is not asking too much at n=19. Any selection should be based on non-biased criteria, such as selecting polymorphisms from various subdomains of the molecule, representing equitably how the changes are distributed over the sequence (in case they do). The authors start with an arbitrary selection of mutations that then reveal channel function different from wildtype. Importantly, there is no unifying direction apparent in terms of the different-from-wildtype phenotype as presented. These data, taken in ensemble, indicate that the interrogated CaV3.2 channel mutations differ from wildtype, for each mutation in a different manner, with two of the mutations showing similar gain-of-function for peak current density. Importantly, the two other mutations do not reveal such a phenotype. It follows that lack of a specific explanatory electrophysiologic phenotype in half of interrogated channel mutations means that the detected gain-of-function in the other two CaV3.2 mutations is simply unclear with regard to their mechanistic impact on trigeminal neuralgia.

Then one of the CaV3.2 gain-of-function channels is directed for expression in adult mouse trigeminal ganglion sensory neurons, evoking hyperexcitablity. However, the authors used mouse trigeminal ganglion sensory neurons, not human neurons, the latter a feasible experimental platform with stronger translational impact, especially for a paper with wider visibility, published in PAIN. The presented result is interesting, but lacking a critical control, namely at least one of the CaV3.2 mutations that did not reveal a gain of function. Importantly, how relevant is the examination of trigeminal ganglion sensory neurons with directed expression of CaV3.2(G563R)? How certain are we that trigeminal ganglion sensory neurons are the critical cellular site for CaV3.2 mutations to contribute to trigeminal neuralgia? Findings in nerve injury, as presented here for infraorbital nerve, or as referenced for DRG-peripheral nerve injury, do not exclude other cellular lineages as critical sites of action for CaV3.2 mutations: glial cells could also be involved such as peripheral glia, namely Schwann cells (ganglionic and peripheral) and satellite cells, and central glia, namely astrocytes, microglia and even oligodendroglia. Besides trigeminal ganglion sensory neurons, there are trigeminal nucleus spinalis pain relay neurons where CaV3.2 is also expressed, possibly also in glial cells that co-function with these neurons. In which cell lineage CaV3.2 expression is critical is simply unknown in trigeminal neuralgia. Last but not least, trigeminal ganglion sensory neurons were derived from “5–7-week-old mice”—of which sex? Directed expression of a human trigeminal neuralgia-associated CaV3.2 mutation to trigeminal sensory neurons is at least as relevant for the experimental objective of Gambeta *et al*. as for their mouse *in-vivo* studies, yet for culture of trigeminal ganglion sensory neurons they were not conducting the experiments in a sex-separate manner as they did for mouse *in-vivo* experiments.

Now, onto the *in vivo* model. The authors have included several qualifier statements why infraorbital nerve constriction injury (CION) is not trigeminal neuralgia. These qualifiers are not wrong, but starting with human genetic polymorphisms that were detected in trigeminal neuralgia, NOT trigeminal neuropathic pain, dictates use of a more validated model, not of a marginally relevant model. Which outcomes of CION research have improved mechanistic understanding and clinical care of trigeminal neuralgia?

Can some cases of familial trigeminal neuralgia be reduced to a CaV3.2 channelopathy? If so, this would allow us to then translate from the infrequent genetic alteration toward the more frequent sporadic disease, sticking with the same target, CaV3.2. The animal data presented by Gambeta *et al*. do not pertain to this concept. They are disjointed because none of the human CaV3.2 mutations were introduced into the mouse, for a dedicated interrogation against a CaV3.2−/− background. As presented, there is simply a complete lack-of-connect between mouse data and human CaV3.2(mutant) physiology data.

Of the mouse data presented, the biggest red flag, peculiarly left uncommented, is that CaV3.2−/− pan-null mice show a highly similar nocifensive behavioral response to CION as do wildtype control mice! Non-injured pain behavior is less sensitive in CaV3.2−/− mice, meaning there is a measurable effect on the trigeminal pain system, depending on presence/absence of CaV3.2. This finding can be interpreted as a “positive control” that the engineered CaV3.2 null mutation affects trigeminal pain processing for one of the measured parameters. But then the pain phenotype in terms of nocifensive behavior in CaV3.2−/− is indistinguishable from wildtype in response to CION!

The documented different response to compound Z944—analgesic in wildtype for 30–60 min, complete lack of effect in CaV3.2−/− mice—should prompt dedicated follow up studies to this interesting starting point, such as sensory neuron-specific knockdown of CaV3.2. But considering this result in the context of the other presented data, it follows that we do not understand trigeminal neuralgia pathophysiology better, and arguably we are also left confused in terms of contributions of CaV3.2 channels to trigeminal peripheral nerve constriction injury.

Moreover, the authors present hypersensitivity caused by CION as measured by heat avoidance. Clinically, in the context of trigeminal nerve pain, cold pain, *e.g.* as can be evoked by the slightest drafts of (cold) air, is a striking hallmark of the human disease which apparently the authors did not consider.

## Data availability

### Underlying data

There are no data associated with this article.
