# Supplementary material for: Mechanistic contribution of CaV3.2 calcium channels to trigeminal neuralgia pathophysiology not clarified
Source: F1000Res. 2022 Jul 27;11:718. Originally published 2022 Jun 29. [Version 2] doi: 10.12688/f1000research.122997.2 (PMC9468618; doi:10.12688/f1000research.122997.2)
Supplement: Peer review history

# Peer review: Reviewer response for version 2

**DOI:** 10.5256/f1000research.136661.r150066

**Published:** 2022-09-12

**Authors:** Kouichi Iwata

**Affiliations:** 1 Department of Physiology, Nihon University School of Dentistry, Tokyo, Japan

The CaV3.2 is the T-type calcium channel expressed in the brain's various neurons and glial cells and involved in the modulation of neuronal excitability and glial cell activation. The CaV3.2 is constructed from multiple subtypes, and these subtypes are involved in different functions in the brain. The CaV3.2 is also engaged in the alteration of nociceptive neuron activity associated with peripheral nerve injury or tissue inflammation. These observations strongly suggest that the CaV3.2 calcium channel contributes to the persistent pain occurrence. The current literature addressed the involvement of the CaV3.2 calcium channel in the trigeminal neuropathic pain mechanism. This literature is exciting and shows us many important issues regarding CaV3.2 in trigeminal persistent pain mechanisms.

 I have just two suggestions in this literature: 1) The CaV3.2 channel is known to interact with various molecules, channels, and receptors. It’s better to state these interactions. 2) If possible, it’s better to address the schematic illustration showing the function of CaV3.2 in trigeminal neuropathic pain.

 I do not have any concerns about indexing this article.

Are arguments sufficiently supported by evidence from the published literature or by new data and results?

Yes

Is the conclusion balanced and justified on the basis of the presented arguments?

Yes

Is the rationale for commenting on the previous publication clearly described?

Yes

Are any opinions stated well-argued, clear and cogent?

Yes

Reviewer Expertise:

NA

I confirm that I have read this submission and believe that I have an appropriate level of expertise to confirm that it is of an acceptable scientific standard.

# Peer review: Reviewer response for version 2

**DOI:** 10.5256/f1000research.136661.r145594

**Published:** 2022-09-01

**Authors:** Nicholas A. Veldhuis, Daniel P. Poole

**Affiliations:** 1 Drug Discovery Biology Theme, Monash Institute of Pharmaceutical Sciences, Monash University, Parkville, Vic, Australia

Prof Liedtke has appropriately addressed our concerns and suggestions. We have no further comments and believe this is a well-written and well-justified correspondence that we hope will influence and inspire pain researchers carrying out similar studies in future.

Are arguments sufficiently supported by evidence from the published literature or by new data and results?

Partly

Is the conclusion balanced and justified on the basis of the presented arguments?

Yes

Is the rationale for commenting on the previous publication clearly described?

Yes

Are any opinions stated well-argued, clear and cogent?

Yes

Reviewer Expertise:

We each have >12 years experience in analgesic drug discovery utilising a range of pre-clinical pain models including migraine

We confirm that we have read this submission and believe that we have an appropriate level of expertise to confirm that it is of an acceptable scientific standard.

# Peer review: Reviewer response for version 1

**DOI:** 10.5256/f1000research.135056.r142603

**Published:** 2022-07-21

**Authors:** Nicholas A. Veldhuis, Daniel P. Poole

**Affiliations:** 1 Drug Discovery Biology Theme, Monash Institute of Pharmaceutical Sciences, Monash University, Parkville, Vic, Australia

This correspondence provides a perspective on a recent publication by Gambeta et al, in the journal PAIN, entitled “CaV3.2 calcium channels contribute to trigeminal neuralgia”.

 Overall, the critique captures and distills all of the key findings made by Gambeta et al. and relates them to a prior human study where polymorphisms in the CACNA1H gene were reported. By doing so it asks readers to consider “what is new” and whether findings in this study progress our mechanistic understanding of how CACNA1H polymorphisms contribute to trigeminal neuralgia.

 The author raises pertinent and valid concerns, within the context of the clinical relevance, which no doubt has limited the mechanistic insight and translational impact of the study. This includes choice of the most appropriate and clinically relevant pain model, rationale behind selection of which missense mutations to assess (i.e. selected sites in only a single domain), sex-specific differences, the methods to assess function (e.g. comparing gain and loss of function on a null background, or lack of a cold pain model) and failure to consider the potential contribution of CaV3.2 expressed by other cell types that drive pain.

 However, recognizing the expectations of a leading specialized journal such as PAIN, and the formative nature of this work, we suggest it would be useful to moderate some of these arguments and provide further constructive feedback, to benefit the authors of this specific study and for future consideration by others attempting related studies: 

1. The comment about justifying the choice of these 4 particular mutants makes sense, but we suggest that author should equally justify screening all 19 mutants by pointing to any related studies in PAIN or equivalent calibre journals where a similarly comprehensive approach has been taken;
2. Comments about the lack-of-connect between mouse data and the human CaV3.2 physiology data would be nicely supported by suggesting logical follow up experiments. This may include assessment using a mouse model expressing the mutant human CaV3.2 or WT human channel, and/or determining specific roles through neuronal vs global deletion.
3. Final paragraph: Rather than stating that the authors simply did not consider the need to comment about the deficiencies of the study with respect to cold pain models, we suggest appending some constructive comments to this paragraph about how the study could have been improved if this had been added.

Are arguments sufficiently supported by evidence from the published literature or by new data and results?

Partly

Is the conclusion balanced and justified on the basis of the presented arguments?

Yes

Is the rationale for commenting on the previous publication clearly described?

Yes

Are any opinions stated well-argued, clear and cogent?

Yes

Reviewer Expertise:

We each have >12 years experience in analgesic drug discovery utilising a range of pre-clinical pain models including migraine

We confirm that we have read this submission and believe that we have an appropriate level of expertise to confirm that it is of an acceptable scientific standard, however we have significant reservations, as outlined above.

# Response

**Published:** 2022-07-24

**Authors:** Wolfgang Liedtke

**Affiliations:** Regeneron Pharmaceuticals, USA

**I am grateful to the reviewer for their careful appreciation of my comment, for their highly informed and constructive feedback.  The reviewer's suggestions have been very helpful for revision of the ms.**

**Specifically, in response:**

1. *"The comment about justifying the choice of these 4 particular mutants makes sense, but we suggest that author should equally justify screening all 19 mutants by pointing to any related studies in PAIN or equivalent calibre journals where a similarly comprehensive approach has been taken;"*
**Three exemplary studies are now referenced.**
2. *"Comments about the lack-of-connect between mouse data and the human CaV3.2 physiology data would be nicely supported by suggesting logical follow up experiments. This may include assessment using a mouse model expressing the mutant human CaV3.2 or WT human channel, and/or determining specific roles through neuronal vs global deletion."*
**The logical follow up experiments are now explicitly spelled out, as suggested.**
3. *"Final paragraph: Rather than stating that the authors simply did not consider the need to comment about the deficiencies of the study with respect to cold pain models, we suggest appending some constructive comments to this paragraph about how the study could have been improved if this had been added."*
**Constructive comments, as suggested by the reviewer, are now appended to this paragraph** **. **
